# Management of Diarrhoeal Dehydration in Childhood: A Review for Clinicians in Developing Countries

**DOI:** 10.3389/fped.2018.00028

**Published:** 2018-02-23

**Authors:** Emmanuel Ademola Anigilaje

**Affiliations:** ^1^Nephrology Unit, Department of Paediatrics, College of Health Sciences, University of Abuja, Abuja, Nigeria

**Keywords:** management, childhood, dehydration, hypovolaemia, isonatraemia, hyponatraemia, hypernatraemia

## Abstract

The survival of a child with severe volume depletion at the emergency department depends on the competency of the first responder to recognize and promptly treat hypovolemic shock. Although the basic principles on fluid and electrolytes therapy have been investigated for decades, the topic remains a challenge, as consensus on clinical management protocol is difficult to reach, and more adverse events are reported from fluid administration than for any other drug. While the old principles proposed by Holliday and Segar, and Finberg have stood the test of time, recent systematic reviews and meta-analyses have highlighted the risk of hyponatraemia, and hyponatraemic encephalopathy in some children treated with hypotonic fluids. In the midst of conflicting literature on fluid and electrolytes therapy, it would appear that isotonic fluids are best suitable for the correction of hypotonic, isonatraemic, and hypernatraemic dehydration. Although oral rehydration therapy is adequate to correct mild to moderate isonatraemic dehydration, parenteral fluid therapy is safer for the child with severe dehydration and those with changes in serum sodium. The article reviews the pathophysiology of water and sodium metabolism and, it uses the clinical case examples to illustrate the bed-side approach to the management of three different types of dehydration using a pre-mixed isotonic fluid solution (with 20 or 40 mmol/L of potassium chloride added depending on the absence or presence of hypokalemia, respectively). When 3% sodium chloride is unavailable to treat hyponatraemic encephalopathy, 0.9% sodium chloride becomes inevitable, albeit, a closer monitoring of serum sodium is required. The importance of a keen and regular clinical and laboratory monitoring of a child being rehydrated is emphasized. The article would be valuable to clinicians in less-developed countries, who must use pre-mixed fluids, and who often cannot get some suitable rehydrating solutions.

## Introduction

The survival of a child with severe volume depletion at the emergency department depends on the competency of the first responder to recognize and promptly treat hypovolemic shock (1). Poor tissue perfusion, acidosis, end-organ damage to the kidneys, liver, and brain, and death are consequences of a poorly managed hypovolaemic child (1–4). In fact, death among volume depleted children with diarrheal disease is the second leading cause of death among the under-five children word wide (5).

Although the basic principles on fluid and electrolytes therapy have been investigated for decades, the topic remains a challenge, as consensus on clinical management protocol is difficult to reach, and more adverse events are reported from fluid administration than for any other drug (6, 7).

While the maintenance fluid therapy promoted by Holliday and Segar in 1957 has stood the test of time (8); recent systematic reviews and meta-analyses have highlighted the risk of hyponatraemia, and hyponatraemic encephalopathy in some children treated with hypotonic fluids that have been used for decades as maintenance therapy (9–13). The compositions of these hypotonic fluids have emanated originally from the Holliday and Segar estimations (8).

Apart from unavailability of some desired fluids (e.g., 3% saline), the clinicians in developing countries are also plagued with dearth of randomized clinical studies on fluids and electrolytes therapy, and confusion arising from conflicting literature on fluids and electrolytes therapy from developed countries, as new research findings continue to confound the old understandings (14–22).

For example, the comprehensive treatise by K. Powers on management of isonatraemic, hypotonic, and hypernatraemic dehydration published in Pediatric Review in 2015 (14) was soon challenged by Moritz and Ayus in 2016 in the same journal (20), with the later authors making the following strong statement; “certain aspects of her (K. Powers) recommendations are incorrect and could pose serious complications if followed.” K. Powers had recommended hypotonic fluids for the treatment of volume-depleted patients, and she had liberalized the use of large quantities of potassium without emphasizing that potassium needs not be administered at a concentration greater than 20 mEq/L unless hypokalemia is present (20). While hypotonic fluids use is associated with a high incidence of hyponatraemia that could result in fatal hyponatraemic encephalopathy; children on high-potassium solutions are also at risk of dangerous hyperkalemia (20).

Furthermore, Kiguli et al. in 2011 (21), following the outcomes of the Fluid Expansion as supportive Therapy (FEAST) study in children with fever and signs of impaired perfusion in African hospitals had concluded that fluid boluses in children with shock were potentially harmful. Nevertheless, the World Health Organization (WHO) in 2013 (22) continued to advocate boluses of isotonic crystalloid as fast as possible to any child fulfilling the WHO definition of shock (i.e., the presence a capillary refilling time of more than 3 s, cold peripheries, a weak pulse, and a fast pulse.). This WHO recommendation made Kiguli et al. to pronounce as follows “the failure of WHO to take account of the FEAST data is disappointing and puzzling, particularly given its commitment to systematic assessment of evidence” (23).

Although the FEAST study excluded children with severe malnutrition and gastroenteritis, and their study population could only meet 2% of the WHO criteria for shock (24); the pervasive undernutrition seen in children of the developing countries would however support the 2013 WHO recommendations on the cautious use of fluid boluses (intravenous fluid is given at a lower volume of 15 mL/kg, over a longer period of 1 h, and if only there is shock), and a slow rehydration over 12 h with oral rehydration solution for malnourished children (ReSoMal) (22) In addition, the existing low oncotic pressure in malnourished children make them leak water into the tissues, with a high chance of dying (25). Thus, the low sodium and low osmolarity in ReSoMal make it safer in malnourished children who because of their high intracellular sodium are prone to retaining fluids, and thus, susceptible to fluid overload and heart failure (26, 27).

Volume depletion denotes reduction of effective circulating volume in the intravascular space, and since sodium constitutes the main extracellular solute, sick children may have isonatraemic, hyponatraemic and hypernatraemic dehydration depending on the differential loss of sodium and water (4, 28). Dehydration denotes lack of plasma free water, and in the strict sense, can only cause hypernatremia (4, 28).

In this article, the terms dehydration and volume depletion will be used interchangeably, even when they are not necessarily the same (4).

The choice of either oral rehydration therapy (ORT) or intravenous (IV) therapy in a child depends on accurate assessment of the degree of dehydration (3). While ORT in a child with an underestimated fluid deficit can cause acidosis, electrolyte disturbances, acute kidney injury, and death; parenteral fluid therapy in a child with an overestimating fluid deficit can also lead to an unwarranted IV interventions, longer hospital stays, fluid overload, and hyponatraemia (3, 29, 30). The importance of close monitoring of a dehydrated child can therefore not be overemphasized, as hydration status can change (i.e., varying degrees of dehydration and over-hydration) even during fluid therapy.

Since pediatric dehydration is frequently the result of gastroenteritis, characterized by vomiting and diarrhea (4), this article will be limited in most part to dehydration resulting from gastroenteritis.

Clinical conditions relating to dehydration from burns, severe malaria, anemia, diabetes ketoacidosis, sickle cell disease, total parenteral nutrition (TPN), severe acute malnutrition, septic shock, acute kidney injury are outside the scope of this article.

Although variations exist in the methods available for assessing dehydration, and in correcting for fluid and electrolytes deficits (1–20), the purpose of this article is to provide a simple, bed-side practical approach in the management of children with hyponatraemic, isonatraemic, and hypernatraemic dehydration. The article goes through the pathophysiology of water and sodium metabolism, and it uses hypothetical clinical case scenarios to illustrate rehydration in children with diarrheal disease presenting with hypotonic, isonatraemic, and hypernatraemic dehydration.

## Terms Relevant to the Discussion

*Diarrhea*: WHO defines diarrhea as the “passage of loose or watery stools at least three times in a 24 h period” but emphasizes the importance of change in stool consistency rather than frequency, and the usefulness of parental insight in deciding whether children have diarrhea or not (31, 32).

*Acute watery diarrhea* refers to diarrhea that begins acutely, lasts less than 14 days, and involves the passage of frequent loose or watery stools without visible blood. Vomiting may occur and fever may be present (31, 32).

*Dysentery* is diarrhea with visible blood in the stools (31, 32).

*Persistent diarrhea* is the diarrhea that begins acutely but is of unusually long duration (at least 14 days). The episode may begin either as watery diarrhea or as dysentery (31, 32) (Table 1).

**Table 1:** Causes of acute and chronic or persistent diarrheal disorders (32).

Types	Examples
Acute diarrhea	Infections Drugs or poisons Immediate onset hypersensitivity reactions

Chronic or persistent diarrhea	Infections with parasites such as cryptosporidium and giardia Other infections, usually in the presence of specific risk factors such as malnutrition, immune deficiency (including HIV, post measles), associated illnesses (pneumonia, urinary tract infections), or mucosal injury Congenital disorders of digestion and absorption including: Exocrine pancreatic insufficiency (e.g., cystic fibrosis); enteropathies (celiac disease, food allergies, autoimmune disorders); specific enzyme defects (sucrase-isomaltase deficiency); transport defects (glucose-galactose transporter); and congenital intractable diarrhea (microvillous inclusion disease, tufting enteropathy) Short gut syndrome (bowel resection after necrotisingenterocolitis)

*Effective circulating volume* denotes the part of the intravascular compartment that is in the arterial system and which effectively perfuse the tissues. Effective circulating volume can be reduced because of *volume depletion* and/or *dehydration* (33, 34).

*Volume depletion* refers to any condition in which the effective circulating volume is reduced (34).

*Dehydration* refers to water loss alone. The consequence of dehydration in the strict sense is hypernatremia (34).

*Hypernatraemic dehydration*: in this type of dehydration, the serum sodium is greater than 145 mmol/L (34, 35).

*Hyponatraemic dehydration*: the condition of hyponatraemia (serum sodium less than 135 mmol/L) in a dehydrated state (34, 35).

*Isonatraemic dehydration*: dehydration is accompanied by normal serum sodium levels between 135-145 mmol/L (34, 35).

*Hyponatraemia* is defined as serum sodium less than 135 mmol/L. It represents an excess of water in relation to sodium in extracellular fluid (ECF) (35).

*Hypernatremia* is when serum sodium is greater than 145 mmol/L. It represents lack of free water in the extracellular space (35).

*Fluid and electrolyte deficit*: a deficit is the amount of water (and electrolytes) lost before rehydrating treatment is begun. For practical purposes, it is a one-time estimate (36).

*Ongoing losses* represent the abnormal losses of fluid and electrolytes that occur after the one-time determination of a deficit. This essentially includes direct measurable losses like diarrhea or vomiting. On-going losses for electrolytes are varied and dependent on the type of fluid being lost and the microbial causes of the diarrhea (36, 37) [Table 2 (37)].

**Table 2:** Average electrolyte content of stool in acute watery diarrhea (37).

Diarrheal by etiology	Sodium mmol/L	Potassium mmol/L	Chloride mmol/L	Bicarbonate mmol/L
**Cholera**				
Children below 5 years	101	27	92	32

**Non-cholera diarrhea**				
Children below 5 years	56	25	55	14

*Mild hypernatraemic* dehydration is when elevated serum sodium is between 146–149 mmol/L (38).

*Moderate hypernatraemic* dehydration is when serum sodium is between 150–169 mmol/L (38).

*Severe hypernatraemic* dehydration is when serum sodium is greater than 169 mmol/L (38).

*Osmolality*: it is defined as the concentration of all the solutes in a given weight of water (34).

*Serum osmolality* (Sosm) is equal to the sum of the osmolality of individual solutes in the intravascular space (34).

*Measured Sosm*: this is Sosm that is measured directly *via* determination of freezing point depression (34).

*Calculated Sosm*: Sosm that is estimated using values of sodium, glucose, and urea in the serum. The serum sodium substantially determines the calculated Sosm.

The formula (34) for calculated osmolality is as follows:

2×sodium (mmol/L)+glucose(mg/dL )/18+blood urea nitrogen (mg/dL)/2.8

*Normal Sosm* is between 275 and 290 mOsm/kg (275–290 mmol/kg) (34).

*The blood tonicity (effective blood osmolality)*: this denotes the concentration of solutes (sodium, glucose, mannitol) that are impermeable to cell membranes which are therefore restricted to the extracellular compartment (33). These solutes are effective because they create osmotic pressure gradients across cell membranes leading to movement of water from the intracellular to the extracellular compartment (33). Since, mannitol is exogenous, the formula (35) for the blood tonicity is therefore:

2×sodium(mmol/L)+glucose(mg/dl)/18.

## The Total Body Water (TBW)

The TBW constitutes 75% of the body weight of the term infant (can be up to 80% in premature infants depending on the gestational age) and decreases to two-thirds of body weight (67%) after the neonatal period. This decreases further to 60% by the age of 12 months, which is the same value we have in adult male (a little less, 55% in adult female) (34).

**Figure 1:**
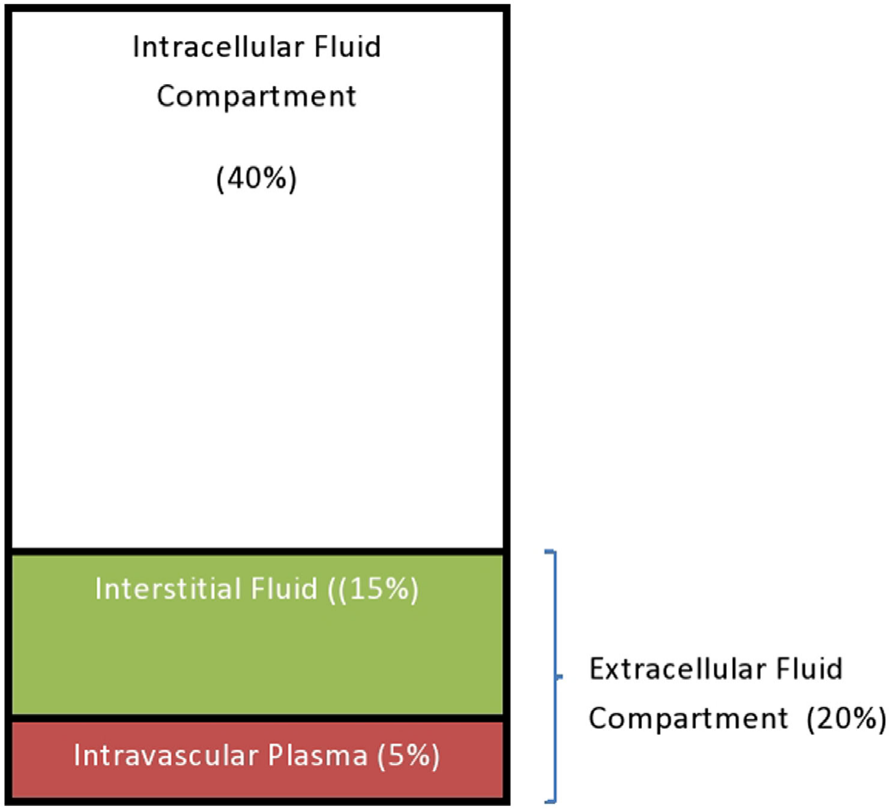
The total water distribution in a 12-month-old child.

The TBW is distributed in various locations throughout the body, known as the fluid compartments. The intracellular fluid (ICF) compartment is about 40% of the TBW. The remaining 20% of the TBW constitutes the ECF compartment. The ECF comprises the interstitial fluid (15% of the TBW) and the intravascular plasma which makes up about 5% of the TBW (34) (Figure 1).

## Regulation of ECF Volume (Sodium and Water Balance)

The ECF volume is determined primarily by sodium and chloride which are by far the most abundant osmotically active solutes in ECF. Since changes in chloride are greatly secondary to changes in sodium, the amount of sodium in the ECF is the most important determinant of ECF volume (39). Table 3 shows the distribution of solutes in the ICF and ECF (35).

**Table 3:** Average solute concentrations in intracellular and extracellular compartments (35).

	Intracellular fluid (mmol/L)	Extracellular fluid (mmol/L)
Cations	Potassium (140) Sodium (13) Mg(7)	Sodium (140) Calcium (2.5) Potasium (4.0) Magnesium (1.1)

Anions	Organic phosphate (107) Protein (40) Bicarbonate (10) Chloride (3)	Chloride (104) Bicarbonate (24) Protein (14) Phosphate (2) Others (6)

Therefore, because of the key position of sodium in volume homeostasis, it is not surprising that more than one mechanism has evolved to control the excretion of sodium. These mechanisms include the glomerular filtration rate (GFR), the juxtaglomerular apparatus, the renin-angiotensin system and others (34, 39) (see Table 4).

**Table 4:** Osmoregulation and volume regulation of extracellular fluid volume (16, 34).

	Osmoregulation	Volume regulation
What is sensed	Plasma osmolality	Effective circulating volume affected by volume depletion and/or dehydration

Sensors	Supraoptic and paraventricular nuclei of the hypothalamus	Cardiopulmonary baroreceptors located in the atria, ventricles and pulmonary interstitium Aortic and carotid baroreceptors Intrarenal receptors in the juxtaglomwerular apparatus and renal intersititium

Effectors	Arginine vasopressin Thirst	Renin-angiotensin-aldosterone Atrial natriuretic peptide Arginine vasopressin

What is effected	Urine osmolality Urine volume Thirst (water intake)	Urinary sodium Thirst (water intake)

Because sodium intake is generally not affected by ECF volume, it means that the sodium in the ECF is controlled by an increase or a decrease in renal sodium excretion (16). The body adjusts to a low ECF volume by making the kidneys to conserve sodium and it responds to a high ECF volume by increasing sodium excretion in the urine (16, 33, 36).

**Figure 2:**
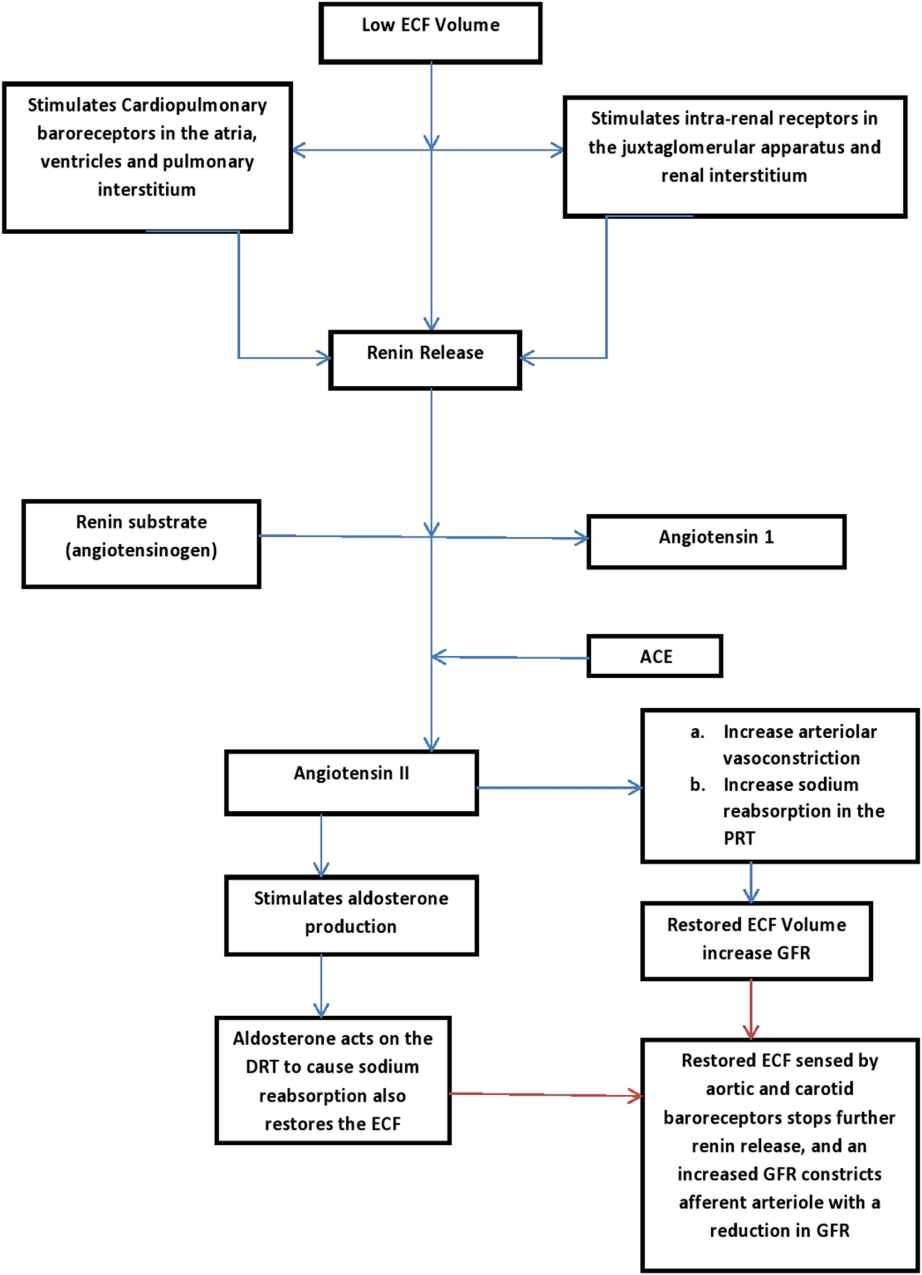
Renin-aldosterone pathways (16, 34). PRT, proximal renal tubles; DRT, distal renal tubles; GFR, glomerular filtration rate; ECF, extracellular volume.

A low ECF volume is detected by cardiopulmonary baroreceptors located in the atria, ventricles, and pulmonary interstitial tissue (34). A low ECF volume is also detected by intra-renal receptors in the juxtaglomerular apparatus and renal interstitium (34). These receptors respond by increasing the renin secretion which in turn leads to activation of the renin-angiotensin-aldosterone system (34). Angiotensin II, the end product of this system is a potent vasoconstrictor, and it causes increased sodium reabsorption in the proximal renal tubules, thereby restoring the blood volume and increasing the GFR (16, 34). Angiotensin II on its own is also a stimulant for aldosterone production. Aldosterone acts on distal tubules to effect sodium reabsorption (34) (see Figure 2 for the pathway of the renin-angiotensin-aldosterone pathway). As the ECF restores back to normal, the increasing GFR initiates a negative tubuloglomerular feedback mechanism that eventually constricts the afferent arteriole with a resultant reduction in glomerular filtration (39). A reduced glomerular filtration translates to reduced filtered sodium that is available for reabsorption at the proximal tubules (34, 39). Hence, the process of sodium reabsorption is halted and a restored ECF terminates the process that is initiated previously (39).

Other mechanisms involved in sodium metabolism include redistribution of intra-renal blood flow and renal prostaglandins (34). The expansion of the ECF volume also increases the secretion of the atrial natriuretic peptide by the heart, and this causes natriuresis and diuresis (34).

Water balance is achieved by ensuring that the amount of water consumed in food and drink and that endogenously generated by metabolism equals the amount of water excreted (34). The consumption side is regulated by behavioral mechanisms including thirst and salt cravings. Water is lost through the skin, lungs, and feces, but the kidneys are the major sites for water regulation (34).

Under normal physiologic conditions, the kidneys control the volume of bodily fluids by the amount of water excreted in the urine. The kidneys conserve water (e.g., in the face of hypovolemia) by producing concentrated urine, relative to plasma, or they can rid the body of excess water by producing diluted urine, relative to plasma.

The direct control of water excretion is exercised by antidiuretic hormone (ADH; now refers to as arginine vasopressin-AVP) (16, 34). AVP is a peptide hormone synthesized in the supra-optic and para-ventricular nuclei of the hypothalamus but stored in the posterior pituitary gland (34). The stimuli for AVP release are increased in Sosm and hypovolemia. However, osmolality is a more sensitive stimulus to AVP secretion as only moderate to severe hypovolemia causes a direct effect on AVP secretion (34). AVP interacts with AVP V2 receptors in the kidney, resulting in the insertion of aquaporin 2 channels on the luminal surface of collecting tubule principal cells (34). As a result, water permeability is increased in the collecting tubules allowing water reabsorption to occur and hence, concentrated urine is produced (increased urine osmolality and decrease urine flow) (16, 34).

Thirst-induced water craving is an important pathologic response to hyper-osmolality or hypovolemia (34). Thirst, however, becomes important when water conservation by the kidneys is inadequate to maintain homeostasis (34). Thirst mechanism is mediated by activation of baroreceptors and the release of angiotensin II (34). The other mechanism that causes thirst is an increase in tonicity (effective osmolality) of ECF, leading to a decrease in ICF, which are sensed by the osmoreceptors located in the anterior hypothalamus (34). Again, osmotic changes are a more effective stimulus of thirst compared with volume changes (34).

When the mechanisms of restoring the ECF volume are compromised, end-organ hypo-perfusion leads to poor oxygenation and nutrient delivery and ultimately tissue acidosis (35, 38).

Table 4 summarizes the mechanisms involved in the regulation of ECF volume (sodium and water balance).

## Isonatraemic Dehydration

In the midst of these compensatory mechanisms, and in the face of ongoing losses, dehydration may ensue, which when accompanied by proportionate loss of sodium from extracellular space results in isonatraemic dehydration. Isonatraemic dehydration is the commonest (70–80%) type of dehydration and the type with the best prognosis (14).

### Diagnosis

In this state, the normal tonicity of body fluids at 275–290 mOsm/kg is maintained, and the serum sodium concentration remains at normal ranges of 135 and 145 mmol/L (34, 38).

### Pathophysiology

The fluid deficit in isonatraemic dehydration is 100% from the extracellular space and when replacing the fluid deficit, the sodium concentration in the repletion fluid should be similar to that of the serum (averages 154 mmol/L) (20, 28, 40). Because of normal Sosm, there is no shift of fluid from ICF to ECF or vice versa.

### Clinical Features

The classical features of dehydration are commonly seen in isotonic dehydration. These include thirst, decreased skin turgor, tachycardia, dry mucous membranes, sunken eyes, lack of tears when crying, a sunken anterior fontanel in infants, and oliguria. The severe features of dehydration include anuria, hypotension, feeble and very rapid radial pulse, cool and moist extremities, and diminished consciousness.

## Hyponatraemic Dehydration

It is seen in 10–15% of cases of dehydration. It occurs more frequently with bacillary dysentery or cholera. Hyponatraemia may develop or worsen if there is a considerable oral ingestion of water, low-electrolyte or electrolyte-free fluids, or diluted formula during diarrhea. It may also follow iatrogenic intravenous administration of hypotonic fluids at the hospital.

### Diagnosis

The serum sodium is less than 135 mmol/L. The Sosm is less than 275 mOsm/Kg because serum sodium accounts essentially for Sosm. Table 5 also outlines other non-diarrheal causes and clinical classes of hyponatraemia (16, 41). A common cause of non-diarrheal hyponatraemia in the hospitalized child that should concern the clinicians is the syndrome of inappropriate secretion of anti-diuretic hormone (SIADH), which diagnosis includes a state of euvolaemia or hypervolemia, hyponatraemia, low urinary output, and inappropriately concentrated urine with elevated urine sodium (41). Cerebral salt wasting (CSW) [seen in intracranial injury, intracranial tumors and neurosurgical procedures] also simulates SIADH. However, in CSW, hyponatraemia occurs in the setting of hypovolemia, normal to high urine output, and a highly elevated urinary sodium excretion (urinary sodium may be >100 mEq/L, far higher than what is seen in SIADH) (41).

**Table 5:** Other non-diarrheal causes, and clinical classes of hyponatraemia (16, 34, 41).

Clinical type	Common causes	Mechanisms of hyponatraemia
Euvolaemia	Syndrome of inappropriate secretion of anti-diuretic hormone (SIADH) Central nervous system (CNS); infections, trauma, surgery, shunts, hypoxic ischemia Pulmonary; pneumonia, effusions, positive pressure ventilation, asthma, tumors Drugs; carbamazepine, vinca alkaloids, narcotics, aspirin, ecstasy, selective serotonin reuptake inhibitors Tumors; leukemia, lymphoma, Neuroblastoma Adrenal insufficiency CNS disease Pulmonary disease Increased water intake; dilute infant formula, polydipsia, near drowning, iatrogenic hypotonic fluid administration	Impaired water excretion (1–4 of the common causes) Increased sodium losses (2, under common causes) Increased water intake (5, under common causes)

Hypervolemia	SIADH Acute/chronic renal failure Nephrotic syndrome Liver cirrhosis	Impaired water excretion (1–4 of the common causes) Increased sodium losses (2 of the common causes)

Hypovolemia	Diarrhea Vomiting Burns Pancreatitis Diuretics Ostomy output Heat stroke Renal tubular acidosis Cerebral salt wasting	Increased sodium losses (1–9 of the common causes)

### Pathophysiology

Since solute loss (in vomiting or diarrhea) almost always occurs in an isosmotic fluid that has a sodium and potassium concentration less than that of plasma, loss of these fluids cannot directly lower the plasma sodium except there is increased water intake, or when excess water is retained (16). Thus, water retention leading to an excess of water in relation to sodium is a common denominator in almost all hyponatraemic states (16). There is often a shift of fluid from the ECF to the ICF due to the predominant loss of ECF, and therefore, vascular collapse requiring urgent circulatory support is seen more often and earlier than in the other types of dehydration (1, 4, 14, 34).

### Clinical Features

Nausea and malaise are typically seen when sodium level acutely falls below 125–130 mmol/L (42). Headache, lethargy, restlessness, and disorientation when sodium concentration falls below 115–120 mmol/L (42). Cerebral edema, seizure, brain stem herniation, respiratory arrest, coma, and death may occur with severe and rapidly evolving hyponatraemia (42).

## Hypernatraemic Dehydration

It is seen in 10–20% of patients with diarrhea. It can occur when too much salt is ingested or due to water deficit. Most frequently, it is due to water deficit from increased water losses in diarrheal stools, and also due to reduced water intake during the illness. In rare cases, it is due to an increase in total body sodium following ingestion of concentrated formula, incorrectly prepared highly concentrated oral electrolyte solution, and administration of high solute containing beverages to a child with diarrhea (41). The potential for hypernatremia also increases with increased evaporative water loss from fever and from high environmental temperature. However, additional free water loss can occur with co-morbidities like diabetes insipidus, renal tubular disorders, or post-obstructive diuresis (41). Reduced water intake is also common in infancy who cannot seek for water to quench thirst or in hypodipsia, when there is hypo-responsiveness to thirst (41).

### Diagnosis

Dehydration state in which serum sodium is greater than 145 mmol/L, and Sosm higher than 290 mOsm/kg (34). Mild hypernatremia is when elevated serum sodium is between 146 and 149 mmol/L (38). Moderate when serum sodium is between 150 and 169 mmol/L, and severe when serum sodium is greater than 169 mmol/L (38).

### Pathophysiology

There is a fluid shift from the intracellular to the extracellular space to maintain intravascular volume (intracellular dehydration), therefore, the vascular volume is usually maintained until the degree of dehydration is quite severe (1, 4, 14). It represents lack of free water in the extracellular space (35). The central nervous system (CNS) features in hypernatraemic dehydration can be explained by two ways depending on whether hypernatremia occurs acutely (within 24–48 h) or chronically (34). In acute hypernatremia, intracellular dehydration of the neurons causes intracranial hemorrhage (subdural, subarachnoid, and intra-cerebral) as water shift rapidly into cerebral vessels causing their over-distention (14). The distended vessels are easily sheared as the brain shrinks (14). In addition, thrombosis of the small veins or dural sinuses can occur (14). The CNS features seen in chronic hypernatremia are due to the development of cerebral edema following attempts to rapidly correct the hyperosmolar state. This is because the neurons are protected from chronic hyperosmolality through the accumulation of osmotically active substances (including taurine, glutamine, glycine, sorbitol, and inositol) termed idiogenic osmoles (14, 34, 35). These idiogenic osmoles act by countering the osmotic forces from the hyperosmolar plasma and therefore protect the neurons from fluid shifts (14, 34, 35). However, if the hyperosmolality is corrected rapidly, idiogenic osmoles that continue to occupy the neurons create a fluid shift from the ECF (lower osmolality after correction) to the ICF, producing cerebral edema (34, 35).

### Clinical Features

Children with hypernatremia tend to have a fever, very dried tongue, doughy feeling skin, and warm extremities (34, 41). The degree of dehydration is often underestimated because the movement of water from the intracellular space to the extracellular space helps to preserve the intravascular volume. Thirst is severe and out of proportion to the apparent degree of dehydration Children may have CNS features that include irritability, high-pitched cry, nuchal rigidity, lethargy, seizures, hypertonia, and coma (41).

## Maintenance Fluid and Electrolytes Needs

The maintenance fluid requirement is the daily water needed for well individual to excrete iso-osmotic urine. The needs for maintenance fluid and electrolytes arose from the need to eliminate heat and solutes (including urea, creatinine, electrolytes, etc.), which are the two by-products of the basal metabolism (8, 36). Majority of the heat generated by the body is lost by evaporation of water from the skin surface and some by the elimination of warmed water vapor from the upper respiratory tract during expiration (8, 36). The losses from these two sites constitute insensible water losses and virtually do not involve solute loss (8, 36). Soluble waste by-products of metabolism could only be excreted in the urine (8, 36).

Because metabolic rate per unit of body weight declines with increasing age, children generate less heat and solute from basal metabolism than do infants and, therefore, require less fluid and electrolytes per unit of body weight (8, 36). While the amount of fluid and electrolytes declines per unit of body weight, it remains constant per kilocalories of basal metabolism (8, 36). Several methods have been developed to explain the maintenance needs in relation to age-related basal metabolic rates (36). These methods include the use of the surface area, the basal calorie and the one proposed by Holliday and Segar in 1957 (8). The surface area method requires a computed table to determine surface area derived from patient’s height and weight, and fluid is administered per square meter of body’s surface area (36). The basal calorie method also requires a table and it involves the most calculations (36).

The Holliday–Segar system has gained wide acceptance and has stood the test of time (8, 36). It is easy to remember and has proven to be sufficiently accurate for most clinical situations (8, 36). Holliday and Segar calculated the rate of caloric expenditure (i.e., the metabolic rate) of hospitalized children and found that it was proportional to the child’s weight (8, 36). The fluid and electrolytes requirements are empirically based on caloric needs of the average hospital patient which approximates the body weight (36).

The Holliday–Segar formula estimates kilocalories that can be equated with milliliters of fluid. For each 100 kcal expended, approximately 100 ml of fluid is required. The 100 ml is a rounded estimate from (a) 50 mL of fluid required to provide for skin, respiratory tract, and basal stool losses, and (b) 55 to 65 mL of fluid required for the kidneys to excrete an ultra-filtrate of plasma at 300 mOsm/L (with a specific gravity of 1.010) without having to concentrate the urine (36).

In Holliday–Segar formula, the caloric expenditure for the *average* child can then be based on body weight (1, 8, 36). See Table 6.

**Table 6:** Holliday and Segar estimation of maintenance fluid (adapted).

Body weight categories	Estimated daily maintenance fluid volume	Estimated fluid rate per hour
Up to 10 kg	100 calories/kg/day or 100 ml/kg/day	4 ml/kg/h

10–20 kg	1,000 calories + 50 calories/kg/day or 1000 ml + 50 ml/kg/day for each kg over 10 kg	2 ml/kg/h

>20 kg	1,500 calories + 20 calories/kg/day or 1,500 ml + 20 ml/kg/day for each kg over 20 kg	1 ml/kg/h

The electrolytes needed for maintenance are based on the electrolyte content of human breast and cow milk and are based on energy expenditure as well (8, 36):

Sodium – 3 mEq per 100 calories
Potassium – 2 mEq per 100 calories i.e., 20 mmol in 1 L
Chloride – 2 mEq per 100 calories

This ordinarily translates to the use of hypotonic fluids containing 1/4 normal saline with 20 mmol/L of potassium chloride or 1/2 normal saline with 20 mmol/L of potassium chloride (20, 36). These fluids will provide the required amount of sodium and potassium necessary to keep their serum concentrations within the normal range in children having no significant additional losses or those with renal impairments. While these hypotonic fluids have been used for decades and have not resulted in hyponatraemia in all patients; a mounting consensus points to a greater risk for hyponatraemia for ill children who are kept on these hypotonic fluids (9–13).

This risk of hyponatraemia arises from impaired excretion of free water in an ill child placed on hypotonic fluids but who already have an increased level of ADH (38). ADH secretion is stimulated by numerous factors and clinical states including pre/post-operative patients, fever, pain/stress, respiratory distress/failure, head trauma, CNS infections, pneumonia, bronchiolitis, hypovolemic states, and exposure to some medications (opiates, SSRIs, NSAIDs, Phenothiazines) (38, 41).

Thus, while hypotonic fluids have a greater risk for hyponatraemia, isotonic fluids for maintenance do not increase the risk of hypernatremia and have proven to be safe (17, 20). Isotonic maintenance fluids do not produce hypernatremia in the absence of a renal concentrating defect or a large extra-renal free water loss, because a normal functioning kidney can generate free water by excreting hypertonic urine (20, 41). Isotonic fluid solutions (0.9% normal saline, Ringer’s lactate, Hartmann’s solution, Plasmalyte 148 solution) are the preferred maintenance fluids for the ill child (38). Refer to Table 7 (43).

**Table 7:** Some examples of hypotonic, isotonic, and hypertonic fluids (16, 44).

Fluid	Na (mmol/L)	Cl (mmol/L)	K (mmol/L)	Ca (mmol/L)	Mg (mmol/L)	Glucose (g/L)	Lactate (mmol/L)^a^	Acetate (mmol/L)^a^	Gluconate (mmol/L)^a^
Hypotonic									
0.18% NaCl	31	31							
0.45% NaCl	77	77	–	–		–			
Isotonic									
0.9% NaCl	154	154							
0.9% NaCl with 5% glucose	154	154				50			
0.9% NaCl with 5% glucose with 20 mmol/L of KCl	154	174	20			50			
Plasmalyte 148 solution	140	98	5		3	–		28	23
Hartmann’s solution	131	111	4	2			29		
Ringer’s lactate	130	109	4	3			28		
Ringer’s acetate	131	109	4	3				28	
Hypertonic									
3% NaCl	513	513							

*^a^Balanced/buffered isotonic crystalloid; Na, sodium; Cl, chloride; K, potassium; Ca, calcium; Mg, magnesium*.

By replacing varying amounts of chloride with lactate (Ringer’s lactate, Hartmann’s solution), acetate (Ringer’s acetate), and with acetate and gluconate (Plasmalyte 148), these isotonic fluids are said to be balanced or buffered as they now have electro-neutrality (balance between positive and negative ions) (44) (Table 7).

Because 0.9% normal saline (an unbalanced/un-buffered isotonic fluid) has been shown to cause dilutional coagulopathy (45, 46), hyperchloraemic metabolic acidosis (45–48), and hyperchloraemic-induced acute kidney injury (49), its use must also be accompanied by close clinical and laboratory monitoring in order to ensure that these complications are prevented and/or are treated promptly once recognized.

However, Ringer’s lactate and Hartmann’s solutions, respectively, contain sodium concentrations of 130 and 131 mmol/L which are relatively lower than the serum sodium and their use should also be closely monitored as they could adversely lower the patient’s serum sodium (14). In addition, Ringer’s lactate should be used cautiously in children with significant emesis, in whom contraction alkalosis (increase in blood pH) could be worsened by the lactate content of Ringers when it eventually got converted to bicarbonate (14).

While the addition of 20 mmol/L of potassium chloride to IV fluid is generally permissible (once a patient has satisfactory urine output to avoid hyperkalaemia), the amount of potassium chloride should be individualized to the patient needs and should be closely monitored (41, 42, 50). Furthermore, in an acutely ill child with nausea and vomiting, the reduced carbohydrate intake leads to free fatty acid breakdown, excess ketones, and an increased likelihood of continued nausea and vomiting. Consequently, adding 5% glucose to an isotonic solution will stimulate insulin release, reduce free fatty acid breakdown, and therefore, reduce treatment failure due to persisting nausea and vomiting (14, 42). The glucose in maintenance fluids also diminish the protein degradation that would occur if the patient receives no calories (51). Glucose also provides added osmoles, thus, reducing the possibility of exposure to hypotonic fluids that may cause hemolysis (51). However, maintenance fluids do not provide adequate calories, protein, fat, minerals, or vitamins, and total parenteral nutrition (TPN) should be provided for children who cannot be fed enterally for more than a few days (51).

Therefore, a preferred fluid solution to be used for maintenance fluid and electrolytes is 5% dextrose in 0.9% saline with 20 mmol/L of KCL (20, 38, 42).

Unless hypokalemia is present, there is no pressing reason to administer more than this quantity of potassium (20, 41).

Another consideration worthy of note in the acutely ill child is that reduction in the excretion of free water makes these children have a reduced urinary output and expectedly, the total fluid required to maintain a normal intravascular volume is reduced (1, 38, 51). If these children receive maintenance IV fluid at standard rates derived from the Holliday-Segar formula, fluid overload may ensue (1, 38, 51).

Hence, while the maintenance volumes should be the starting point for these acutely ill dehydrated children, adjustment (between 50 and 80% reductions in maintenance volumes) can be made based on the clinical response to fluids in these children (2, 38).

In contrast, some sick children will have increased fluid requirements (e.g., those with high fever, capillary leak, third spacing of fluid into the abdomen or those with continuing losses). These children may need more than the standard maintenance fluid rate to maintain normal intravascular volume (38, 51). Refer to Table 8 for conditions requiring limiting or increasing the maintenance fluid volumes.

**Table 8:** Conditions requiring limiting or increasing the maintenance fluid volume (38).

Conditions when maintenance therapy is decreased	Conditions when maintenance therapy is increased
Postoperative children	Increased activity
Children with brain or lung disease(meningitis, encephalitis, bronchiolitis, pneumonia) and other hospitilized children	Fever
Coma	Burns
Hypothermia	Excessive sweating
Hypothyroidism	Tachypnea
Oliguria/anuria	Tachycardia
Highly humidified atmospheres	Dry environment
Humidified ventilator circuits	Hyperventilation
	Capillary leak
	Third spacing of fluid
	Extreme low birth weight
	Use of overhead heaters
	Use of phototherapy units
	Polyuria
	Surgical drains

Above all, prescribing maintenance fluid for ill children requires regular assessments, including weight assessment in order to ensure that adequate hydration is achieved without putting children to hyponatraemia and or over-hydration (38, 51).

## Assessing Fluid Deficit/Volume Depletion

A deficit is the amount of water (and electrolytes) lost before treatment is begun (36). For practical purposes, it is a one-time estimate, and additional abnormal losses are considered in the category of on-going losses, which together with the fluid deficit must be corrected before a child could maintain a normal ECF status (36, 52).

Although acute weight change (each gram of weight loss corresponds to 1 mL of water loss) is the most direct basis for determining the amount of deficit fluid, however (36), this must be considered carefully as an acute weight change may not occur in a volume depleted individual who is retaining fluid in the “third space” (36).

From the foregoing, it is admissible that assessing the extent of volume depletion can be difficult. This difficulty is also compounded by the fact that in most cases, the pre-illness weight of a child is unknown and the clinician is constrained to use clinical signs and symptoms as well as laboratory data to assess the degree of dehydration (1, 4, 36). Over the years, the constellations of these signs and symptoms have been made into scales for scoring the degree of dehydration. Examples include those of the WHO (53) (Table 9), the 4 and 10-point Gorelick Scale (54) (Table 10) and the Clinical Dehydration Scale (54) (Table 11).

**Table 9:** World Health Organization scale for dehydration for children 1 month to 5 years old (53).

	A	B	C
Look at condition	Well, alert	Restless, irritable	Lethargic or unconscious
Eyes	Normal	Sunken	Sunken

Thirst	Drinks normally, not thirsty	Thirsty, drinks eagerly	Drinks poorly or not able to drink

Feel; skin pinch	Goes back quickly	Goes back slowly	Goes back very slowly ≥ 2 s

*Scoring; fewer than two signs from column B and C; no signs of dehydration, <5%; ≥2 signs in column B; moderate dehydration, 5–10%, ≥2 signs in column C; severe dehydration, >10%*.

**Table 10:** Gorelick scale for dehydration used in children between 1 month and 5 years (54).

	No or minimal dehydration	Moderate to severe dehydration
*General appearance*	Alert	Restless, lethargic, unconscious
*Capillary refill*	Normal	Prolonged or minimal
*Tears*	Present	Absent
*Mucous membranes*	Moist	Dry, very dry
eye	Normal	Sunken, deeply sunken
breathing	Present	Deep, deep and rapid
Quality of pulses	Normal	Thread, weak or implapable
Skin elesticity	Instant recoil	Recoil slowly; recoil > 2 seconds
Heart rate	Normal	Tachycardia
Urine out put	Normal	Reduced, not passed in many hours

*4 point scale, italics*.

*≥2 clinical signs (4 point) ≥ 5% body weight from baseline*.

*≥3 clinical signs (4 point) ≥ 10% body weight from baseline*.

*10 point scale (all signs/symptoms)*.

*≥3 clinical signs ≥ 5% body weight from baseline*.

*≥7 clinical signs ≥ 10% body weight from baseline*.

**Table 11:** Clinical dehydration score for children 1 month to 3 years (54).

	Score of 0	Score of 1	Score of 2
General appearance	Normal	Thirsty, restless or lethargic but irritable when touched	Drowsy, limp, cold, sweaty ± comatose

Eyes	Normal	Slightly sunken	Very suncken

Mucous membrane	Moist	Sticky	Dry

Tears	Tears	Decreased tears	Absent tears

*A score of 0 indicates no dehydration, 1–4 indicates mild-to-moderate dehydration, and 5–8 indicates severe dehydration*.

Generally, regardless of what scoring method is used, the severity of dehydration is classified as mild (3–5% volume loss), moderate (6–10% volume loss), or severe (9–15% volume loss) (36). From the first principle of a higher TBW in an infant, the higher % values (5 to 10 to 15) are used for an infant and the lower values (3 to 6 to 9) for an older child (36, 55).

Infants and children with mild dehydration often have minimal or no clinical changes other than dry mucous membranes (36). Early hemodynamic signs of intravascular depletion (e.g., tachycardia) define moderate dehydration; and signs of more profound hypovolemia (e.g., hypotension, poor perfusion) are considered evidence of severe dehydration (36). Children with severe dehydration can also present in near-shock to shock with a change in sensorium (lethargy), tachycardia, hypotension, hyperpnea, prolonged capillary refill, and cool and mottled extremities and aggressive isotonic fluid resuscitation are imperative (42). It is important to remember that hypotension is a very late sign of dehydration and it occurs when all compensatory mechanisms to maintain organ perfusion are overwhelmed.

In the replacement for fluid deficit, it is important to remember that each gram of weight loss equals I milliliter of fluid (36), therefore, if an infant weighing 10 kg (10,000 *g* = 10,000 ml) is clinically estimated to be mildly dehydrated at 5%, the volume of deficit to be given is 500 ml.

For a simple bedside calculation, a formula is easily derivable to give this estimate as follows; % dehydration × 10 × body weight (in kg).

Traditionally, intravenous fluids to replace existing fluid deficit are given over the first 24 h, while the patient continues to get his maintenance fluid over the same 24-h period (42).

However, many clinicians quickly administered the fluid deficit either in whole (100%) in the first 8 h (i.e., sequential deficit/maintenance approach) or in part (i.e., half in the first 8 h followed by the remaining half over the subsequent 16 h) as a combined deficit/maintenance approach (36, 55). In both approaches, the patient continues to get his daily maintenance fluid and fluids are also replaced for the on-going losses as well (36, 55).

The appropriate fluid to correct fluid deficit is isotonic fluid with glucose and KCl if required, (once urine output is established). A good example is 0.9% normal saline with 5% glucose with 20 mmol/L of KCl added (up to 40 mmol/L of KCL can be given when there is a proven hypokalemia) (14, 20). This fluid is suitable for isonatraemic, hypernatraemic, and hyponatraemic deficits (14, 20, 38, 42). The only exception is when there is hyponatraemic encephalopathy (with seizures or decreased conscious state) when hypertonic saline (3% NaCl) is preferred in the first few hours of fluid therapy (20).

While Moritz and Ayus have argued strongly against the use of isotonic saline in treating hyponatraemic encephalopathy (because “it is of insufficient tonicity to consistently increase the plasma sodium and could lead to a fall in plasma sodium if AVP concentrations are persistently elevated”); normal saline has been inevitably used when 3% NaCl is not available (14, 41). This may be the case in most hospitals in Africa.

The clinician must remember that the clinical assessment of dehydration is only an estimate; therefore, the child must be continually re-evaluated during therapy to ensure that adequate replacement volumes are administered.

## Replacing On-Going Fluid and Electrolyte Losses

On-going losses represent fluid and electrolytes that are being lost, and which causes the state of volume depletion in the first place (vomiting and diarrhea from gastroenteritis) (36) Therefore, in addition to providing fluids and electrolytes to meet maintenance and deficit needs, on-going losses must be replaced to achieve euvolaemia (14, 36, 51). The on-going losses generally should be replaced milliliter-for milliliter with fluids that have the same electrolyte composition as the on-going losses. For example, the components of gastric fluid include sodium of 60 mmol/L, potassium of 10 mmol/L, chloride of 90 mmol/L, and the preferable repletion fluid is 0.45% normal saline with 10 mmol/L of KCL or oral rehydrating solutions (ORS; replaced at a volume of 2 ml/kg/episode of vomiting if estimating the volume of vomitus is difficult or impossible) (14, 51).

Non-cholera diarrhea fluid contains sodium of 56 mmol/L and chloride of 95 mmol/L, cholera stool contains a higher sodium of 101 mmol/L, and chloride of 92 mmol/L. (Table 2) Although the old ORS developed by the WHO had both higher osmolarity and sodium contents than the new WHO hypo-osmolar solutions (Table 12), data in adults suggest that both formulations are equally effective in cholera but that hyponatraemia, albeit asymptomatic, are seen more frequently in patients given the hypo-osmolar solution (31, 56). However, more information on the use of reduced osmolarity ORT in children with cholera is needed (31).

**Table 12:** Compositions of some common oral rehydration solution-ORS (16, 55, 56).

	Sodium mmol/L	Potassium mmol/L	Base mmol/L	Glucose mg/dl	Osmolality mmol/L
Old World Health Organization (WHO) ORS	90	20	30	2.0	310
New WHO ORS	75	20	10	4.2	245
Pedialyte	45	20	30	2.5	270
Ricelyte	50	25	34	3.0	290

Diarrheal stool can also be replaced with 10 mL/kg of ORS per episode of diarrheal stool or the rate of replacement can occur cumulatively every 1–6 h (14, 51).

## Management of a Child with Dehydration

**Figure 3:**
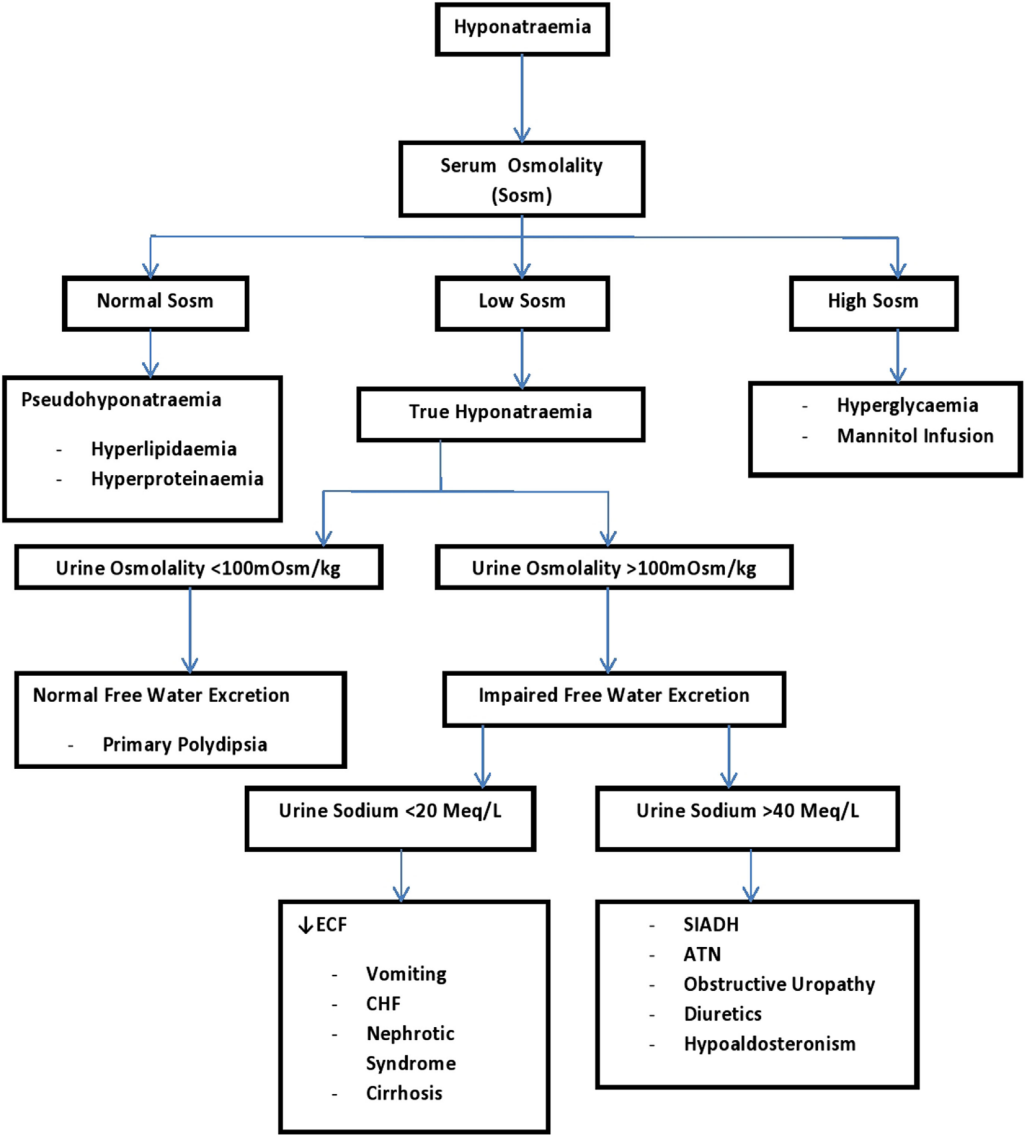
A systematic approach to a patient with hyponatremia using urine sodium and urine osmolality (34). ATN, acute tubular necrosis; CHF, congestive heart failure; ECF, extracellular fluid; SIADH, syndrome of inappropriate antidiuretic hormone.

The management of a child with dehydration must seek to answer the following questions (55):

1. Volume deficit/degree of dehydration. The clinician must first determine if hypovolaemic shock is present (this always signifies severe dehydration) and must be treated as an emergency. A careful history and further physical examination can point to the possible types of dehydration (41). Determining the degree of dehydration is necessary for the estimation the volume of fluid required to correct the dehydration, and it also determines if rehydration will be by oral route (mild to moderate dehydration) or parenteral (severe dehydration) (14). Paying attention to the intravascular volume should tell if the patient is hypovolaemic, euvolaemic, or hypervolaemic as this becomes important in the management approach of a child with hyponatraemia (Table 5) (41). However, most patients with hypernatremia have some degree of ECF volume compromise except for patients with mild forms of diabetes insipidus who are able to maintain TBW by responding to thirst stimulation (34, 52). In the process of rehydrating the child the “A” (airway), “B” (breathing), and “C” (circulation) approach still follow. Hypoxia worsens cerebral edema, hence pulse oximetry should be monitored and hypoxia must be corrected by 100% oxygen administration (42, 52). The fluid therapy addresses the circulation and close clinical observation is required to check the patient’s progress to fluid and electrolytes therapy. The steps 2–5 below are particularly important in all children with moderate to severe dehydration.
2. Does osmolar disturbance exist? While it is not important to do elaborate laboratory investigations for mild dehydration, it is important to obtain the serum sodium, Sosm, urinary osmolality, urinary sodium, fractional excretion of sodium, and the urinary specific gravity for moderate to severe forms of dehydration (34). Serum glucose estimation is important in an un-well child who has been eating poorly and in those with unexplained hyper osmolality with hyponatraemia (34).
  In patients with hypernatremia, the normal response is an increase in ADH release, resulting in an increase in urinary osmolality that can reach up to 500 to 1200 mOsm/kg. The presence of this increase in urinary osmolality suggests that the water loss responsible for the hypernatremia (in the absence of osmotic diuresis) is coming from extra-renal (skin, gastrointestinal, and pulmonary) sources. In comparison, hypernatremia with urinary osmolality below 300 mOsm/kg is indicative of renal concentration defect. This could be due to diabetes insipidus (ADH deficiency or non-response to ADH) or other renal concentration defects seen in the polyuric phase of acute tubular necrosis and diuresis that may follow a relief of urinary obstruction (posterior urethral valves). The distinction between central (ADH deficiency) and nephrogenic (non-response to ADH) diabetes insipidus can be made with the administration of desmopressin acetate (DDAVP), an analog of ADH. If the urine osmolality rise by at least 50% in the first 2 h of DDAVP (with a concomitant fall in urine volume), this has proven a state of ADH deficiency (i.e., central diabetes insipidus) (16, 34).
  In hyponatraemia, clinical evaluation of the intravascular volume, Sosm, urine osmolality, and urinary sodium are helpful for the specific diagnosis of the hyponatraemia (34). See Figure 3 for the step-wise approach in the clinical evaluation of a dehydrated child with hyponatraemia who may have other co-morbidities.
3. Check for acid-base disturbance by determining the blood pH, the serum PCO2 and the serum bicarbonate (38, 55). There could be normal anion gap metabolic acidosis from loss of bicarbonate in diarrhea stool, increased anion gap metabolic acidosis from ketoacidosis (lipolysis following poor oral intake) and lactic acidosis (poor tissue perfusion) (38). There could be compensatory respiratory alkalosis as well (38). Children with serum HCO3 < 17 mEq/L can either have moderate or severe hypovolemia but never mild hypovolemia (55)
4. Check the serum potassium. Serum potassium may be low following the loss in diarrheal stool (1). It may be high following shifts from intracellular to the extracellular compartment with metabolic acidosis, when renal failure sets in, and following improper administration of therapy fluid containing potassium (1, 14). It may also be low as metabolic acidosis is reversed. Therefore, low, normal, or high serum potassium should be interpreted cautiously as it may not be reflective of the total body potassium.
5. Renal function test. The kidney may fail following tissue hypo-perfusion resulting from prolonged uncorrected hypovolemia (55). It is important to assay for the serum urea and creatinine, and diagnose renal failure when it ensues. Urinary sodium and fractional excretion of sodium can help to quickly distinguish pre-renal hypo-perfusion from an established renal failure (14). A fractional excretion of sodium less than 1% suggests a pre-renal or hypovolemic state that should respond to volume replacement (14). The fluid therapy must follow that of a renal failure once it is diagnosed.

## Treatment of Diarrheal Dehydration

It is important to emphasize that oral rehydration can be safely and effectively accomplished in children with mild-to-moderate dehydration and in those with normal serum sodium values. Children with more severe dehydration or with abnormal serum sodium values should be treated with intravenous infusions (14, 38, 41, 42). Most oral rehydration solutions contain sodium concentrations in the range of 50–90 mmol/L (Table 9), which are lower than the serum sodium and their use could adversely lower the patient’s serum sodium. They can lower the serum sodium further in hyponatraemic dehydration and can lower the serum sodium greater than 0.5 mmol/L per hour in hypernatraemic dehydration (38). In the hyponatraemic child with neurological changes or seizure (and if the serum sodium is less than 120 mmol/L), urgent repletion of sodium is needed which oral rehydration may not be able to provide (38). Therefore, caution must be applied in the assumption that most cases of dehydration (including children with mild to moderate hyponatraemic or hypernatraemic dehydration) can be managed with oral rehydration (52). Children with severe volume depletion, especially those with hypernatremia or hyponatraemia require inpatient therapy (4), and the offer of ORT may reduce the importance of the need for the clinician to closely monitor these groups of children (1).

The following discussion focuses on how to treat the different types and severity of dehydration that may be seen in diarrheal disease, and it also emphasizes on the necessary intensive monitoring required in all types of dehydration:

### Oral Rehydration for Mild to Moderate Isonatraemic Dehydration

Oral rehydration therapy either by mouth or through the nasogastric tube can safely and effectively restore intravascular volume in children with mild-to-moderate isonatraemic dehydration, in as much as the child can tolerate it (1). The American Academy of Pediatrics, WHO, and center for disease control advocate for ORT as first-line therapy for mild and moderately dehydrated children (1, 57–59). A systematic review and meta-analysis found no clinical difference in rehydration among children with oral versus intravenous fluids and concluded that ORT should be the first-line therapy for mild-to-moderate dehydration, with intravenous therapy used only if oral therapy fails (14).

Oral rehydration therapy works in the most severe form of diarrhea. Even with on-going diarrhea, water can be absorbed across the intestinal lumen by the co-transport of sodium and glucose via the SGLT1 protein and by active transport via the sodium-potassium ATPase pump (14). Optimal glucose transport at concentrations: glucose 2–2.5 gm/L and Na 45–90 mEq/L (55).

The glucose: sodium ratio of rehydration fluids should not exceed more than 2:1 for effective fluid repletion (1). Higher glucose concentration exacerbates diarrhea and sodium loss, and hence, the need for new WHO-ORS (55). While clinical aspects of diarrheal illness do not permit a specific etiological diagnosis to be made with confidence and stool microscopy or culture is not a routine practice; however, cholera should be suspected in children >2 years old who have acute watery diarrhea, with signs of severe dehydration or shock, especially when there is suspected epidemic of cholera in the locality.

Beverages like apple juice, tea, ginger ale, colas, sports drinks, and chicken broth are inappropriate to use for rehydration because they do not contain the correct sodium and glucose ratio to promote salt and water reabsorption (1, 14, 55).

Oral rehydration therapy is unsuitable in the following conditions; (a) severe dehydration, (b) circulatory instability or hypovolemic shock, (c) altered mental status, (d) intractable vomiting, (e) bloody diarrhea, (f) paralytic ileus/abdominal distension, (g) abnormal serum sodium values, (h) glucose mal-absorption, (i) lack of caregiver to give close attention during ORT, and in (j) those averse to the taste of oral rehydration solution or those not tolerating because of stomatitis or pharyngitis (1, 14).

ORT involves the following:

1. The amount of fluid deficit is calculated based on the change in weight (if pre-illness weight is known) or based on clinical signs. Generally, mild to moderate dehydration is assumed at 5% (50 ml/kg body weight) and this is replaced over 2–4 h (1, 14). Using a teaspoon, syringe, or dropper, 1–2 ml/kg should be administered every 5 min (or 5–10 mL ever 5–10 min), with the volume increased as tolerated, especially in children with nausea and vomiting. Nasogastric tubes can also be used in patients with severe vomiting or oral ulcers (1, 14).
2. Replace ongoing losses with ORS. When possible and measurable, ongoing losses should be replaced volume for volume using appropriate fluid, or with ORS. When it is impossible to have a measurement of the vomitus or stool, replacing 10 mL/kg body weight for each watery stool or 2 mL/kg body weight for each episode of emesis should suffice (1, 14).
3. Breastfeeding/formula or age-appropriate complementary feed should not be stopped during ORT as they serve as maintenance. Small frequent feeds decrease vomiting. However, carbonated drinks and drinks or food with high concentrations of simple carbohydrates should be avoided during therapy, and patient should be reassessed regularly (1, 14, 60).
4. Pharmacologic Therapy. The common causes of infectious diarrhea are shown in Table 13. There are also variations in the microbial causes of diarrhea between developed and developing countries. In developed countries, about 70% of diarrhea cases are of viral (40% rotavirus), 10–20% of bacterial, and <10% of protozoal origin (61–64). In developing countries, 50–60% of cases are of bacterial (Enteropathogenic *E. Coli* 25%, *Campylobacter jejuni* 10–18%, *Shigella* spp, and *Salmonella* spp 5% each), 35% of viral (15–25% rotavirus) origin, and in many the cause is unidentified or mixed (61–65). Therefore in developing countries where bacterial causes are more common, local and WHO guidelines for antimicrobials should be followed (1). Bacterial cause of diarrhea is more likely in the presence of high fever, bloody diarrhea, mucoid diarrhea, and severe abdominal pain (59).
  Newer antiemetic like ondansetron is favored as it decreases vomiting during rehydration, it reduces the need for intravenous therapy (IV) and hospital admission, and it does not have the extrapyramidal effects of promethazine (66, 67). The main concern with the use of ondansetron is cardiac arrhythmias, especially in children with long QT syndrome (68). The oral dose is as follows; 8–15 kg: 2 mg; 15–30 kg: 4 mg; >30 kg: 8 mg (max dose). IV dose is 0.1–0.5 mg/kg/dose (max 4 mg) in a child who is not tolerating the oral dose (69). There is no role for antidiarrheal agents. Vitamin A and Zinc are also useful supplements in the management of diarrhea in infants and children (70).
5. Challenges in the administration of ORT include the perception that IV therapy is superior to ORT; ORT is time-consuming for both parents and doctors; parents expect IV therapy once they are on admission at the hospital; and parents expect IV therapy since they may have tried ORT at home, even when they have used poor technique (1).
6. Some key measures to prevent diarrhea include: access to safe drinking-water, improvement in environmental sanitation, exclusive breastfeeding for the first 6 months of life, good personal, and food hygiene (hand washing), health education about how infectious diarrhea is spread and administration of rotavirus and typhoid vaccines to susceptible children (1).

**Table 13:** Common pathogen causing childhood diarrhea (61–64).

Etiology	Common pathogens
Viruses	Rota virus Noro virus Enteric adenovirus Other; caliciviruses, astroviruses, enteroviruses

Bacteria	*Campylobacter jejuni* Non-typhoid *Salmonella* spp Enteropathogenic *Escherichia coli* *Shigella* spp *Salmonella typhi* Shiga-toxin producing *Escherichia coli* *Vibrio cholerae*

Protozoa	*Cryptosporidium parvum* *Giardia lamblia* *Entameba histolytica*

Un-identified	
Mixed infections	

### Treatment of Hypovolemic Shock in Severe Dehydration (Isonatraemia, Hyponatraemia, or Hypernatremia)

Once it is determined that a patient is in shock, the general principles of hypovolemic shock management per pediatric advanced life support should be followed including obtaining rapid vascular access, which may be in the form of peripheral intravenous access or an intraosseous route if peripheral access is not successful (42, 71). This also includes the administration of a high concentration of oxygen (ensuring that 100 percent of the available arterial hemoglobin is oxygenated) (42, 71).

**Hypothetical clinical case 1. Management for severe isonatraemic dehydration**

AB, a 11- month old child presented at emergency unit with 3 days history of vomiting and diarrhea, physical examination revealed that the child was lethargic, dried mouth, tearing, sunken eyeballs and anterior fontanelle, pulse was rapid and weak, BP was 45mmHg/inaudible diastolic capillary refills was in excess of 4 s. Child is making urine, but mother has no need to change the nappies as she usually does before the illness. The child weighs 8 kg. The laboratory results revealed Na 138 mmol/L, K 1.8 mmol/L, Cl 95 mmol/L

❖ What type and degree of dehydration is AB?
❖ Outline the fluid prescription for AB?

AB has severe isonatraemic dehydration with hypokalemia. AB also presented in hypovolaemic shock, so rapidly expand the intravascular space with 0.9% Normal saline, i.e., 20 mL/kg = 160 mL, given over 10 min as initial fluid therapy.

Calculate the deficit at 15% because AB is an infant, i.e., 15 × 10 × 8 = 1,200 ml; however, 160 mL has been given as a volume expander, you are left with 1,040 mL as a remaining deficit volume.

Calculate the maintenance fluid volume, i.e., 100 × 8 = 800 mL

Measure ongoing loss by possible recording volume of stool or vomitus and record the volume to be replaced volume for volume with ORS. If measurement is impossible give 10 mL/kg = 80 mL of ORS/loose stool and 2 mL/kg = 20 mL of ORS/vomitus

You can either use the combined deficit/maintenance approach OR sequential deficit-maintenance approach

❖ **For combined deficit/maintenance approach:**

Give 1/2 remaining deficit (1/2 × 1,040 = 520 ml) + 1/3 maintenance (1/3 × 800 = 267 mL) = 787 mL in the first 8 h

Then, follow with 1/2 remaining deficit (1/2 × 1,040 = 520 mL) + 2/3 maintenance (2/3 × 800 = 533) = 1,053 ml over the remaining 16 h

❖ **For sequential deficit/maintenance approach:**

Give 1,040 mL (remaining deficit) over the first 8 h

Give 800 maintenance fluids over the remaining 16 h

Choice of fluid for both maintenance and deficit is Isotonic Saline (0.9%) with 5% dextrose

20 mmol of KCl into each liter of fluid could have been adequate but could be increased to 40 mmol/L because of the hypokalemia (1.8 mmol/L). Once the serum K rises to 3.5 (i.e., hypokalemia corrected) decrease KCL to 20 mmol/L.

Regular monitoring is required

Hypovolaemic shock must be treated once recognized regardless of whether the child is having isonatraemia, hyponatraemia, or hypernatremia (20, 38, 42). Clinical signs of shock or hypovolemia are poor peripheral perfusion, cool pale extremities, tachycardia with low volume pulses, impaired consciousness, high blood lactate, or large base deficit. Hypotension is often a late sign. Excluding those with SAM, suspected malaria or anemia, children with more than one of these signs should be given 20 mL/kg of IV Normal saline over 5–10 min or as rapidly permitted by the vascular access. Up to 60 mL/kg (i.e., 3 boluses of 20 mL/kg) may need to be given within the first hour before the plasma volume is restored. Any child requiring more than 60 mL/kg in fluid boluses should be carefully reviewed to consider the need for a vasopressor or inotropic support (Septic shock) (41, 71). Patient should be monitored closely for lung edema as the resuscitative fluid is given. Use of hyponatraemic fluids is forbidden. Potassium is also not given at this phase (41).

### Parenteral Fluid Therapy for Severe Isonatraemic Dehydration Including Provision for Deficit Volume, Maintenance Fluid, and On-Going Losses

Stages involved (14, 36, 55):

1. Initial fluid therapy/resuscitative fluid (i.e., Treatment of hypovolemic shock). Remember that this is part of deficit replacement.
2. Subsequent therapy: replace deficit (remember that the initial fluid therapy for hypovolemic shock is part of the deficit volume and this volume should be accounted for in the total volume of calculated deficit volume) + maintenance fluid volume + on-going losses.
3. Final therapy: when patients return to normal composition/establish oral feeds/correct potassium deficit
4. Monitor therapy continuously.

**Hypothetical clinical case 2. Management of asymptomatic hyponatraemic dehydration with serum sodiumabove120 mmol/L**

SR, an 8 week old infant weighing 4 kg presented with 1 week history of vomiting and passage of loose stool. Mother was advised to give water as frequently as the child could tolerate it. He was found with moderate dehydration on examination. Laboratory results revealed Na 130 mmol/L, Cl 94 mmol/L, K 1.9 mmol/L, and HCo3 8 mmol/L.

Deficit fluid volume: 10 × 10 × 4 = 400 mL

Maintenance fluid volume: 100 × 4 = 400 mL

Measure ongoing loss by possible recording volume of stool or vomitus and record the volume to be replaced volume for volume with ORS. If measurement is impossible give 10 mL/kg = 40 ml of ORS/loose stool and 2 mL/kg = 8 ml of ORS/vomitus

❖ **For combined deficit/maintenance approach:**

Give 1/2 deficit (200 mL) + 1/3 of maintenance (133 mL) in the first 8 h, then 1/2 of remaining deficit (200 mL) and 2/3 of the remaining maintenance (267 mL) in the next 16 h

Regular monitoring is required

❖ **For sequential deficit/maintenance approach:**

Give 400 mL of deficit volume in the first 8 h

Give 400 mL of maintenance fluid in the next 16 h

❖ Choice of fluid for both maintenance and deficit is Isotonic (0.9%) normal saline with 5% dextrose

20 mmol of KCl into each liter of fluid could have been adequate but this could be increased to 40 mmol/L because of the hypokalemia (1.9 mmol/L). Once the serum K rises to 3.5, i.e., hypokalemia corrected) decrease KCL to 20 mmol/L

Regular monitoring is required.

### Parenteral Fluid Therapy for Hyponatraemic Dehydration Including Provision for Deficit Volume, Maintenance Fluid, and On-Going Losses

For the most part, the following discussion will focus more on the management of hyponatraemia in a child with volume depletion.

Hyponatraemia is seldom symptomatic unless the serum Na is < 120 and if the hyponatraemia occurs quickly (41). So, therefore, if Serum Na is lower than 135 mmol/L but higher than 120 mmol/L, and the child is not convulsing, then management is essentially like that of isonatraemic dehydration.

**Hypothetical clinical case 3. Management of symptomatic hyponatraemic dehydration, i.e., serum Na less than 120 mmol/L and the child is convulsing**

JK is a 5 month old child brought to the emergency unit with a 7 day history of vomiting and diarrhea. JK had stopped breastfeeding in the last 48 h which made JK’s mother to institute tea and water which JK took poorly. 15 min before presentation, JK was said to have convulsed, generalized tonic-clonic which lasted for 15 min. On examination, JK was found with dried tongue, sunken eyes and depressed anterior fontanel and tenting of the skin, weight was 5 kg, pulse rate was 160 beat/min, cold and pale extremities. The blood pressure was 45 mmHg/inaudible diastolic. Laboratory evaluation revealed serum Na of 105 mmol/L, CL 95 mmol/L, K 3.5 mmol/L, serum HCO3 8 mmol/L. Outline the management approach of JK.

JK is having severe hyponatraemic dehydration with serum Na < 120 mmol/L, and metabolic acidosis.

Since JK is in shock give 20 mL/5 kg = 100 mL of Normal Saline.

Calculate JK fluid deficit at 15% deficit = 15 × 10 × 5 = 750 mL

Actual deficit left is (750–100) = 650 mL

Calculate JK Na deficit = 120–105 × 0.6 × body weight = 15 × 0.6 × 5 = 45 mmol of Na

This is equal to 90 mL of 3%NaCl, since 0.5 mmol of Na = 1 ml of 3% NaCl which should be given over 30 min. This dose can be repeated until the serum sodium becomes 120–125 mmol/L.

*(If 3% NaCl is unavailable, the 45* *mmol of Na can also be gotten from 292* *mL of 0.9 saline, since 0.154* *mmol of Na* = *1 ml of 0.9% NaCl)*.

Calculate JK maintenance fluid volume = 100 × 5 kg = 500 mL

Give the remaining deficit, 560 mL (650–90 mL) to go in the first 8 h

Give the maintenance of 500 ml to go over the remaining 16 h

It only makes sense that only the sequential deficit/maintenance approach can be used if there is a need to correct symptomatic hyponatraemia

The fluid choice for the deficit and maintenance volume is isotonic saline (0.9%) with 5% glucose and the rate of rise in serum sodium should be monitored hourly as the serum sodium should not rise more than 0.5 mmol/L every hour or 12 mmol/L over 24 h (i.e., from 120 to 132 mmol/L)

Once child is making urine, add 20 mmol/L of KCL into isotonic saline with 5% dextrose.

Regular monitoring is required.

The initial goal in treating hyponatraemia is the correction of intravascular volume depletion with isotonic normal saline. Urgent treatment of hyponatraemia is however required in all patients who exhibit neurological changes including seizure, change in sensorium and if the serum sodium level is < 120 mmol/L (41). Most frequently, hypertonic 3% NaCl (513 mmol/L) is used and should be given through a central venous line although careful administration through a peripheral IV or intra-osseous needle is acceptable in an emergency (31). The goal is to raise the serum sodium to 120–125 mmol/L or until the seizure stops (41). To raise the serum sodium level, the calculated amount of hypertonic saline is given over 15–30 min to gain rapid control of the seizures (41). Obviously, the rapidity (over 30 min) with which the 3% NaCl needed to be given means that the otherwise combined deficit-maintenance methods are unsuitable in this case. The approach is to calculate the Na deficit that must first be given acutely as part of the fluid deficit. The patient also gets his maintenance fluid and the on-going losses are also replaced.

The formula for the Na deficit mmol = (120–current Na) × 0.6 × body weight in kg (41).

The 0.6 is 60%, which is the volume of distribution of sodium. The mmol of Na required should ideally be gotten from 3% NaCl as it contains 513 mmol of Na/L or 1 mL = 0.5 mmol of Na. A rule of thumb is that 4 mL/kg of 3% NaCl increases serum Na by 3 mmol/L (38, 41).

Although isotonic 0.9% saline is inappropriate for the treatment of hyponatraemic encephalopathy because it is insufficiently hypertonic to result in a consistent increase in plasma sodium; it may still be given if a hypertonic saline solution is unavailable, 1 mL of 0.9% saline contains 0.154 mmol of Na (14, 41). This translates to a higher volume which is also given as part of deficit volume (14).

Acute rapid correction of hyponatraemia to stop seizures or address neurological changes followed by a slower correction (10–12 mmol/L/day) to normal serum levels is essential to prevent central pontine myelinolysis (CPM). CPM although rare in children, is characterized by neurologic symptoms including confusion, agitation, flaccid or spastic quadriparesis, and death. CPM is also commoner in patients treated for chronic hyponatraemia than in those treated for acute hyponatraemia.

### Parenteral Fluid Therapy for Hypernatraemic Dehydration Including Provision for Deficit Volume, Maintenance Fluid, and On-Going Losses

Hypernatremia (serum sodium > 145 mmol/L) usually arises because too much of free water is lost which should be replaced gradually. It is moderate hypernatremia when serum sodium is 150–169 mmol/L and severe hypernatremia when serum sodium is > 169 mmol/L.

The gradual rehydration protocol suggested by Finberg in 1973 remains valid up till today (72).

After the administration of resuscitative isotonic saline for hypovolemic shock (in hypernatraemic dehydration, as in any dehydration, the first priority is the restoration of intravascular volume with isotonic fluid), the volume of free water deficit should be calculated and given slowly over 48 h in moderate hypernatremia and over 72 h in severe hypernatremia (38).

At both rates (48 or 72 h) of rehydration, the caution is not to reduce the serum sodium by more than 0.5 mmol/L per hour and/or by more than 12 mmol/L over 24 h (1, 38, 52). Close monitoring of the rate of decrease of the serum sodium concentration permits adjustment in the rate of fluid that the patient is receiving, in order to avoid the overly rapid correction of the hypernatremia (52).

It is the free water deficit volume that is spread over 48–72 h as the child continues to get his daily maintenance fluid and fluid is continuously being replaced for ongoing losses (1, 38, 52).

The deficit volume in hypernatraemic dehydration is the free water and the formula for free water deficit is (1, 41):

$$\text{Free water deficit}(\text{in L})=\frac{\left[\text{current Na level in mmol}/\text{L}-1\right]}{145\text{ mmol}/\text{L}}\times 0.6\times \text{weight }(\text{in kg})$$

Ideally, the best fluid to give is water as it contains 100% free water (52). 0.45% normal saline and 0.225% normal saline contain 50 and 75% free water, respectively (41, 52). 1 L of 5% dextrose in 0.45% normal saline will provide 400 mL of free water (41, 52). 1 L of 0.9% isotonic saline provides no free water (52). The dilemma is that the more the solution contains free water, the higher the risk of the patient developing hyponatraemia during therapy. The caution is, therefore, to expand the ECF first with isotonic saline, after which, 0.45% normal saline can then be used.

**Hypothetical clinical case 4. Management of severe hypernatraemic dehydration, i.e., serum Na >169 mmol/L and the child also in hypovolaemic shock**

TP is a 3 month old child who presented at emergency pediatric unit (EPU) with 3 weeks history of fever and occasional vomiting. TP mother also noticed that the child has not been suckling well as before and she also felt light in her breasts. TP mother complained to her neighbor who advised her to buy infant formula as the child “may not like her breast milk again” TP mother was adding little water to many scoops of the infant formula, contrary to the instruction on the tin. However, TP continues to get worse and she was rushed to the EPU by 3 am. The house officer could not understand why the mother was worried as examination revealed only a dried tongue and doughy feeling skin. The weight was 5 kg. The house officer treated TP for malaria fever and asked the mother to come back later in the day. He also requested for E/Urea because of the history of vomiting. The result came out 1 h later revealing malaria parasitaemia of 1 + , Na of 170 mEq/L, K 3.7 mEq/L, Cl 104 mEq/L. When the senior doctor examined the child later, TP was confirmed to be in hypovolemic shock.

The child had severe hypernatraemic dehydration and also in hypovolemic shock

So 20 mL/kg of NS was given over 20 min = 100 mL

Calculated free water deficit = (170/145−1) × 0.6 × 5 = 0.52 L = 517 mL

The remaining deficit = 517 – 100 = 417 mL

The calculated maintenance = 100 mL × 5 = 500 mL

Child received (1/3 of 417 mL) 139 mL of deficit + 500 mL of maintenance = 639 mL in the first 24 h (+ongoing losses)

Another (1/3 of 417) 139 mL of deficit + 500 mL of maintenance = 639 mL in the next 24 h (+ongoing losses)

Remaining (1/3 of 417 mL) 139 mL of deficit + 500 mL of maintenance = 639 mL in the next 24 h (+ongoing losses)

In other word, the free water deficit was spread over 72 h (i.e., severe hypernatremia) from the time of treatment commencement, while the child continued to receive his daily maintenance and ongoing losses

The fluid choice is isotonic saline (0.9% NaCl with 5% glucose)

20 mmol/L of KCL was added

|Regular monitoring is required.

**Regular monitoring:**

It is important to remember that the bed-side assessment for dehydration remains at best clinical estimates; therefore, close monitoring of patients on rehydration is the key. In as much as the kidney is functioning optimally, errors of under and over estimation in the calculation of fluid estimates are often corrected by the kidney. While the cause of the dehydration is being treated and regardless of the type of dehydration, the clinician should pay attention to the following (36, 41, 52);

- Regular monitoring of the vital signs (respiratory rate, pulse, and blood pressure, Po2) and having a good record of the fluid intake and output (Fluid balance, urine output) and close watch on the physical examination (weight, clinical signs of depletion or overload). The best monitoring “tests” are the simple ones that all too frequently are overlooked, such as the resolution of signs of dehydration apparent on physical examination (i.e., tachycardia, abnormal respiratory pattern, dry mucous membranes, etc.)
- Children receiving deficit fluid should be weighed at least every 6 h
- A daily increase in weight of 5% or more indicates fluid overload, provided there is no third-spacing. Manage by stopping IV fluids and measure serum sodium
- A daily decrease in weight of 5% or more indicates dehydration. Pay a closer attention to fluid rates, to ensure adequate rehydration
- Daily assess for edema (eyelid and lower limb swelling) and stop IV fluid if edema is identified
- Check serum electrolytes and glucose daily
- Check serum electrolytes and glucose every 4 h, if there is hypo/hypernatraemic dehydration, until serum values become normal
- Red flag if serum sodium is ≤ 130 mmol/L or has fallen by > 0.5 mmol/L per hour or >12 mmol/L per day OR serum sodium is ≥ 150 mmol/L or has risen by > 0.5 mmol/L per hour or >12 mmol/L per day
- If sodium is correctly too rapidly in hyponatraemic dehydration, cerebral (especially pontine) demyelination, and permanent brain injury may occur
- If sodium is corrected too rapidly in hypernatraemic dehydration, cerebral edema may occur
- The closure of rehydration therapy is suggested by normalization of vascular status, restoration of normal mental status, lack of clinical signs, and symptoms of fluid deficit, and adequate urinary output of >0.5 mL/kg/h (1 mL/kg/h for an infant).
- Other endpoints include normalization of laboratory results including blood urea, serum creatinine, Sosm, urine osmolality, and urinary electrolytes.
- Normalization is impossible if ongoing losses continue and this should be corrected as well.

Again, it appears that isotonic 0.9% normal saline with 5% dextrose is an appropriate fluid, and it is safe as a starting fluid in cases of hypernatraemic dehydration (1, 20, 38, 42, 52).

Even though the deficit in hypernatremia is predominantly water, it is safe to use isotonic saline (0.9% with 5% glucose) for both maintenance and deficit volumes as isotonic fluids are superior to hypotonic fluids for the prevention of hyponatraemia and are not associated with an increase in the incidence of hypernatremia or fluid overload (10, 14).

However, the clinician must ever remain vigilant and adjust appropriately the rate (reduce by 20%) of rehydration with isotonic saline as the reduction in serum sodium should not be more than 0.5 mmol/L per hour (28, 41, 52).

Management of hyponatraemia involves the following:

Give bolus of 0.9% saline 20 mL/kg if child is in shock and as required. In the child with hypernatraemic dehydration, as in any child with dehydration, the first priority is restoration of intravascular volume with isotonic fluid

Calculate fluid deficit=% deficit×weight (kg)×10

Subtract the bolus given for hypovolaemic shock from calculated deficit = remaining deficit

Calculate maintenance fluid based on weight of the child

For mild/moderate hypernatremia;

Give 1/2 of remaining deficit over the first 24 h + 24 h maintenance fluid + ongoing losses
Give 1/2 of remaining deficit over the second 24 h + 24 h maintenance fluid + ongoing losses

For severe hypernatremia;

Give 1/3 of remaining deficit over the first 24 h+ 24 h maintenance fluid + ongoing losses
Give 1/3 of remaining deficit over the second 24 h + 24 h maintenance fluid + ongoing losses
Give 1/3 of remaining deficit over the third 24 h + 24 h maintenance fluid + ongoing losses

The fluid of choice for correcting hypernatraemic dehydration is 0.9% NaCl with 5% glucose

## Conclusion

The management of a child with dehydration requires a regular and close clinical and laboratory monitoring. In as much as the kidneys are functioning optimally, errors resulting from over-hydration can be corrected by the kidneys. Onus is on the attending physician to ensure that hypovolaemic shock is reversed, while ongoing losses are replaced to remove the child from a perpetual state of dehydration.

## Clinical Pearls

- A preferred fluid solution to be used for maintenance fluid and electrolytes is 5% dextrose in 0.9% saline with 20 mmol/L of potassium chloride added.
- The maintenance fluid volumes are the starting point for acutely ill dehydrated children, adjustment (between 50–80% reductions in maintenance volumes) can be made based on the clinical response to fluids in these children who tend to have a reduced free water excretion from high levels of ADH hormones. Some children may, however, require more than the usual maintenance fluid volumes.
- Maintenance fluid containing less than 75 mmol/L of sodium should never be used for maintenance hydration. 0.18% sodium chloride (so-called paediatric saline) contains only 30 mmol/L of sodium and is not appropriate for maintenance hydration.
- Oral rehydration is most suitable for mild to moderate isonatraemic dehydration.
- 0.9% saline with 5% glucose with 20 mmol/L of potassium chloride (up to 40 mmol/L of potassium chloride can be given when there is a proven hypokalemia) is suitable for most isonatraemic, hypernatraemic, and hyponatraemic dehydration.
- The correction of the deficit-free water volume in hypernatraemic dehydration is spread over 48–72 h, as the child continues to get his daily maintenance fluid, and fluid is continuously being replaced for ongoing losses.
- If sodium is corrected too rapidly in hypernatremia, cerebral edema, and permanent brain injury may occur.
- If sodium is corrected too rapidly in hyponatraemia, cerebral (especially pontine) demyelination, and permanent brain injury may occur.
- Regular clinical and laboratory (serum electrolytes and glucose) monitoring every 4 h is required in hyponatraemic and hypernatraemic dehydration until serum values become normal.
- The rate of change in serum sodium should not be more than 0.5 mmol/L per hour and/or 12 mmol/L per day in hyponatraemic and hypernatraemic dehydration.

## Author Contributions

Responsible for the conceptualization, literature review, and critical review of the manuscript.

## Disclaimer

While the information contained in this article is scientifically correct, the author hereby disclaims any and all liability to all persons arising out of, or related to the content of this article. Readers are advised to seek the services of an experienced, competent professional if expert assistance is required in childhood fluid and electrolytes therapy.

## Conflict of Interest Statement

The author declares that the research was conducted in the absence of any commercial or financial relationships that could be construed as a potential conflict of interest.
